# Evaluating multiple spatial scales to understand the distribution of anuran beta diversity in the Brazilian Atlantic Forest

**DOI:** 10.1002/ece3.2852

**Published:** 2017-03-12

**Authors:** Lara G. Melchior, Denise de C. Rossa‐Feres, Fernando R. da Silva

**Affiliations:** ^1^ Programa de Pós Graduação em Biologia AnimalUniversidade Estadual Paulista Júlio de Mesquita Filho – UNESPSão José do Rio PretoSão PauloBrazil; ^2^ Departamento de Zoologia e BotânicaUniversidade Estadual Paulista Júlio de Mesquita Filho – UNESPSão José do Rio PretoSão PauloBrazil; ^3^ Laboratório de Ecologia Teórica: Integrando Tempo, Biologia e Espaço (LET.IT.BE), Departamento de Ciências AmbientaisUniversidade Federal de São Carlos – UFSCarSorocabaSão PauloBrazil

**Keywords:** dispersal limitation, environmental heterogeneity, nestedness, species replacement, stochasticity, tadpoles

## Abstract

We partitioned the total beta diversity in the species composition of anuran tadpoles to evaluate if species replacement and nestedness components are congruent at different spatial resolutions in the Brazilian Atlantic Forest. We alternated the sampling grain and extent of the study area (among ponds at a site, among ponds within regions, among sites within regions, and among sites within regions pooled together) to assess the importance of anuran beta diversity components. We then performed variation partitioning to evaluate the congruence of environmental descriptors and geographical distance in explaining the spatial distribution of the species replacement and nestedness components. We found that species replacement was the main component of beta diversity, independent of the sampling grain and extent. Furthermore, when considering the same sampling grain and increasing the extent, the values of species replacement increased. On the other hand, when considering the same extent and increasing the sampling grain, the values of species replacement decreased. At the smallest sampling grain and extent, the environmental descriptors and geographic distance were not congruent and alternated in the percentage of variation explaining the spatial distribution of species replacement and nestedness. At the largest spatial scales (SSs), the biogeographical regions showed higher values of the percentage explaining the variation in the beta diversity components. We found high values of species replacement independently of the spatial resolution, but the processes driving community assembly seem to be dependent on the SS. At small scales, both stochastic and deterministic factors might be important processes structuring anuran tadpole assemblages. On the other hand, at a large spatial grain and extent, the processes restricting species distributions might be more effective for drawing inferences regarding the variation in anuran beta diversity in different regions of the Brazilian Atlantic Forest.

## 1 Introduction

Total species richness of a region, frequently named gamma diversity (γ), can be partitioned in two components: alpha diversity (α) that is the number of species by site, and beta diversity (β) that is the variation in the species identities from site to site (Whittaker, 1960, 1972). The concepts of beta diversity and species turnover have often been used interchangeably in the ecological literature; however, the failure to recognize the distinction between these terms can lead to the inappropriate use of some beta diversity indices (Anderson et al., 2011; Koleff, Gaston, & Lennon, 2003). Recently, Baselga (2012) partitioned the total beta diversity into two components, nestedness and species replacement. Nestedness is observed when the species composition of sample units with low richness represents a subset of the species found in the richest sample units (Baselga, 2010, 2012). This beta diversity component represents the gain or loss of species in communities without replacement. The main assumptions underpinning the nestedness distribution are related to different habitat characteristics (size, isolation, heterogeneity, and quality) and some attributes of species (regional abundance, minimum area requirements, niche breadth; see Ulrich, Almeida‐Neto, & Gotelli, 2009). On the other hand, species replacement involves species turnover as a result of species sorting, stochastic events, geographic barriers, and/or biogeographical regions involving more than one regional species pool (Gaston, Evans, & Lennon, 2007; Leibold et al., 2004; Svenning, Floigaard, & Baselga, 2011). Although it is recognized that the spatial distribution of beta diversity is related to processes and mechanisms operating at different spatial scales (SSs; Chase, 2014; Kirchheimer et al., 2016; Levin, 1992; Nekola & White, 1999; Wiens, 1989), few studies have evaluated congruence in the distribution of beta diversity considering similar SS in different regions (Comte, Monier, Crevecoeur, Lovejoy, & Vincent, 2016; Olivier & van Aarde, 2014).

**Figure 1:**
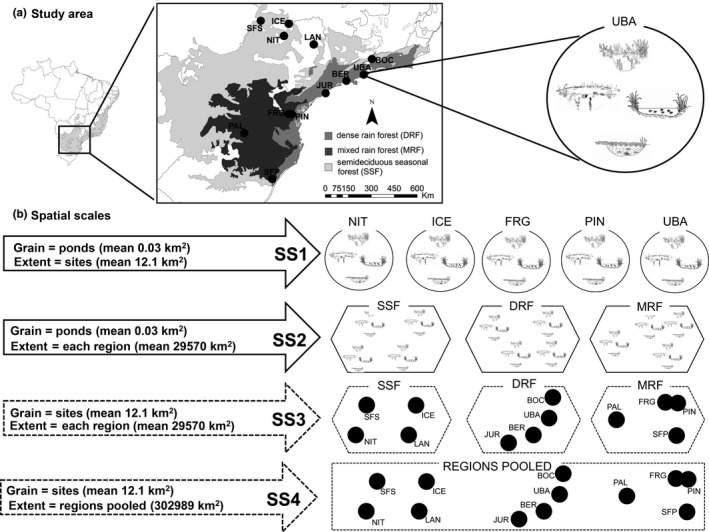
(a) Original Brazilian Atlantic Forest distribution and the 12 sites evaluated in this study. Forest types are indicated by different shades of gray (light gray—semideciduous seasonal forest—SSF, gray—dense rain forest—DRF, and dark gray—mixed rain forest—MRF). Ubatuba (UBA) is highlighted illustrating that different ponds were sampled within sites. (b) Schematic representation of the different spatial scales addressed in this study. Arrows with solid lines consider ponds as the sampling units and the sites (SS1) or the forest types (SS2) separately as the extent. Arrows with dashed lines consider sites as the sampling units and the forest types separately (SS3) or the three forest types pooled together (SS4) as the extent. Circles represent sites, hexagons represent each region separately, and rectangle represents regions pooled. Details of the sites are in Appendix S1

**Figure 2:**
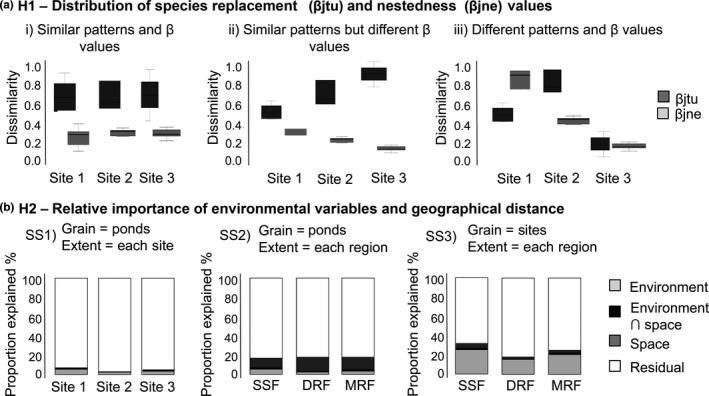
Illustration of the hypotheses evaluated in this study. (a) Three scenarios for the distribution of species replacement (βjtu) and nestedness (βjne) values considering ponds as sampling unit and sites as extent (SS1): (i) Species replacement is the main beta diversity component in the three sites and dissimilarity values are similar among sites; (ii) species replacement is the main beta diversity component in the three sites, but dissimilarity values are different among sites; (iii) species replacement and nestedness values are dependent on the site and dissimilarity values are different among sites. For illustrative purpose we showed SS1, but it can be applied to all spatial scales. (b) Predictions of the relative importance of environmental variables and geographical distance explaining variation in anuran community composition at different spatial scales. Please see text to details of the predictions

Here, we partitioned the total beta diversity of the species composition of anuran tadpoles to evaluate if species replacement and nestedness distributions are congruent at different spatial grains and extents across the Brazilian Atlantic Forest. This biome is home to approximately 600 species of amphibians, of which approximately 73% are endemic (Haddad, Toledo, Prado, Loebmann, & Gasparini, 2013). Recently, Vasconcelos, Prado, da Silva, and Haddad (2014) proposed that the species composition of anurans in the Brazilian Atlantic Forest can be split into four regions that are broadly congruent with the vegetation formations of the Atlantic Forest: (1) Region 1, located in Atlantic Forest inland areas, encompasses most of the semideciduous forest and transitional areas to the Cerrado; (2) Region 2 comprises the coastal Atlantic Forest in southeastern Brazil, where most of the area falls within the ombrophilous forest; (3) Region 3 is mostly congruent with the Araucaria forest in southern Brazil; and (4) Region 4 encompasses the northeastern Brazilian semideciduous and ombrophilous forests. Based on this classification, we explored the community similarity of anuran species at multiple SSs (among ponds at a site, among ponds within regions, among sites within regions, and among sites within regions pooled together; Figure 1). Our first objective was to evaluate whether species replacement and nestedness values are congruent considering similar SSs within and among regions of the Brazilian Atlantic Forest (Figure 2a). This approach will help us to understand if distribution patterns of beta diversity obtained in one study apply only to the area under investigation or whether they can emerge on other communities considering similar SSs (Lawton, 1999). Our second objective was to understand if ecological processes such as species sorting and dispersal limitation are congruent within and among different regions considering similar spatial grains and extents. To this, we evaluated four different SSs across the Brazilian Atlantic Forest (Figure 1):

SS1) Beta diversity among ponds within each site (smallest spatial grain and extent): At small grain, both stochastic species occupancy among sampling units and deterministic variation in species responses to habitat heterogeneity could determine the spatial distribution of beta diversity (Chase, 2007; Vellend, 2016; Vellend et al., 2014). Studies conducted in tropical and temperate regions have found that anuran species richness is positively correlated with the quantity of vegetation and/or diversity of vegetation types within ponds (Burne & Griffin, 2005; da Silva, Gibbs, & Rossa‐Feres, 2012; Hazell, Hero, Lindenmayer, & Cunningham, 2004). However, species occurring in the ponds with low environmental heterogeneity are not subsets of species occurring in ponds with high environmental heterogeneity (Vasconcelos, Santos, Rossa‐Feres, & Haddad, 2009). Furthermore, each pond contains fewer species than the total species richness observed in sites, indicating that ponds differ in species composition (see Table S1 in Appendix S1). Based on these facts and considering that the smaller the grain, the greater the dissimilarity among the sampling units (Nekola & White, 1999), we predict high values of species replacement among ponds because of the variation in stochastic factors such as recruitment or random colonization (Chase, 2007; Hubbell, 2001). If the values of species replacement are similar among sites, we expect that all sites will present higher values of species replacement than nestedness (Figure 2a), in all regions sampled. Furthermore, if stochastic factors are the main drivers of the species replacement, we expect no association with environmental descriptors or geographic distance (Figure 2b);
SS2) Beta diversity among ponds within each region (smallest spatial grain and intermediate extent): Compared to SS1, we increased the extent from sites to regions. Because we increased the regional species pool when the extent was increased (Harrison & Cornell, 2008), we predict that the values of species replacement among the ponds will be higher than the values observed in SS1 (Barton et al., 2013). Because community similarity decays with distance (Nekola & White, 1999), we expect that geographic distance will have a greater relative importance in determining the spatial distribution of species replacement than local environmental descriptors (Tuomisto, Ruokolainen, & Yli‐Halla, 2003; Figure 2b);
SS3) Beta diversity among sites within each region (intermediate spatial grain and intermediate extent): Compared to SS2, we increased the grain from ponds to sites. An increase in the grain generally decreases the dissimilarity among the sample units because a greater proportion of the spatial heterogeneity of the system is contained within the grain (Barton et al., 2013; Wiens, 1989). Thus, the regional species pool is similar to that of SS2, but we increased the number of species within a single sample unit (Nekola & White, 1999; Wiens, 1989). Because sites within the same region are influenced by similar climatic conditions and regional species pool (da Silva, Almeida‐Neto, Prado, Haddad, & Rossa‐Feres, 2012), we predict that the differences in species composition among the sites will be due to turnover of rare anuran species. Therefore, we expect higher values of species replacement than nestedness;
SS4) Beta diversity among sites among the three regions pooled together (intermediate spatial grain and largest extent): Compared to SS3, we increased the extent from each region to the regions pooled together. An increase in the extent generally increases the dissimilarity among the sample units by including different biogeographical areas (Wiens, 1989). At this large spatial extent, the variation in species is associated with historical and evolutionary events (e.g., speciation and extinction), geographical barriers, and environmental filters (Harrison & Cornell, 2008; Svenning et al., 2011). Because regions contain different regional species pools (Vasconcelos et al., 2014), we predict that the values of species replacement will be lower among sites within the same region than among those of different regions. Therefore, we expect that the values of species replacement will be associated with the region in which sites are located due to environmental filters and/or dispersal limitations (da Silva, Almeida‐Neto, & Arena, 2014; da Silva, Almeida‐Neto, et al., 2012).

## 2 Materials and Methods

### 2.1 Study area

The Brazilian Atlantic Forest hotspot is one of the most diverse biomes in the world (Mittermeier, Myers, Mittermeier, & Robles Gil, 2005). Its broad geographical variation ranging from latitudes of 6°N to 30°S and longitudes of 35°W to 52°W results in a climatic gradient related to the annual rainfall (from approximately 800–4,000 mm) and mean annual temperatures (averages from 15 to 25°C), which influence floristic distributions (Oliveira‐Filho & Fontes, 2000). According to Oliveira‐Filho and Fontes (2000), the south and southeast Brazilian Atlantic Forest can be classified into three forest types: (1) dense rain forest (hereafter DRF)—this forest is associated with the Atlantic coast, with elevations ranging from 50 to 2,200 m a.s.l. It occurs in climates with high and constant rainfall throughout the year that ranges from 2,000 to 3,600 mm (Oliveira‐Filho & Fontes, 2000). The annual mean temperature (AMT) varies between 22 and 25°C (Colombo & Joly, 2010); (2) semideciduous seasonal forest (SSF)—this forest is associated with inland areas with elevations below 700 m a.s.l. It occurs in climates with a prolonged dry season (from 2 to 6 months—from April to September). SSF has an annual rainfall that ranges from 1,500 to 2,000 mm (Oliveira‐Filho & Fontes, 2000) and an AMT that varies between 22 and 25°C (Colombo & Joly, 2010); and (3) mixed rain forest (MRF)—this forest occurs in the southern Atlantic Forest, with a northern distribution limit in the Serra da Mantiqueira (latitude 20°S) at elevations above 500 m a.s.l. It occurs in areas subjected to tropical and sub‐tropical humid climates without pronounced dry periods. MRF has an annual rainfall that ranges from 1,400 to 2,200 mm and temperatures that vary from 12 to 18°C (Colombo & Joly, 2010; Oliveira‐Filho & Fontes, 2000). Duarte, Bergamin, Marcilio‐Silva, Seger, and Marques (2014) found that MRF contain different lineages when compared to DRF and SSF likely resulting from the biogeographical origin of several taxa occurring in these forests. According to these authors, MRF are related to conifers, while DRF and SSF are related to Myrtales and fabids, respectively. The vegetation types of the Atlantic Forest (Oliveira‐Filho & Fontes, 2000) are congruent with regions based on anuran species composition proposed by Vasconcelos et al. (2014). Therefore, for this study we considered the names of vegetation formations (SSF, DRF, and MRF) for the broadest scale (Figure 1).

### 2.2 Anuran tadpole data and spatial scales

We compiled distributional records of tadpole assemblages (presence and absence data) from literature and data from the project SISBIOTA CNPq/FAPESP Brazilian Tadpole Biology (coordinate by Denise C. Rossa‐Feres). These studies were carried out with standardized surveys across the DRF, SSF, and MRF regions in the Brazilian Atlantic Forest. We limited our study to three of the four regions proposed by Vasconcelos et al. (2014) because there are no checklists of tadpole assemblages that encompass the northeastern Brazilian Atlantic Forest. To reduce potential bias, we selected only studies that (1) sampled tadpoles with a wire mesh dip net; (2) carried out the surveys during the rainy season, which is the reproductive period of most anuran species, and (3) carried out the surveys in ponds, puddles, or marshes (hereafter ponds), excluding streams and other lotic systems. We obtained tadpole assemblages for 102 ponds (38 in SSF, 41 in DRF, and 23 in MRF) distributed across 12 sites (see Table S1 in Appendix S1; Figure 1). Overall, we gathered 96 anuran species with SSF, MRF, and DRF regions harbored 32, 34, and 52 species, respectively, and four anuran species occurred in all three regions (see Table S2 in Appendix S1).

Based on these data, we used different spatial grains (i.e., ponds and sites) and extents (i.e., sites, each region separately, and regions pooled together) across the Brazilian Atlantic Forest to evaluate the congruence in the distribution of beta diversity considering four SSs (Figure 1).

### 2.3 Environmental descriptors of sampling units

The environmental descriptors of the ponds were obtained from original studies (see Table S1 in Appendix S1). They were selected based on preview studies that demonstrated the importance of these descriptors for the species richness and composition of anurans (da Silva, Gibbs, et al., 2012; Hecnar & M'Closkey, 1998; Van Buskirk, 2005). The environmental descriptors selected were (1) hydroperiod: classified as permanent or temporary; (2) pond area: considering the maximum pond width and length (in m^2^); (3) maximum depth (in meters); (4) pond location: inside forest, at forest edge, or open area; (5) number of vegetation types on the pond margins; and (6) number of vegetation types in the interior of the pond: Both were scored as one of four categories: (1) no vegetation, (2) only herbaceous vegetation, (3) herbaceous vegetation and shrubs or trees, and (4) herbaceous vegetation, shrubs, and trees.

The climatic descriptors of the sites were extracted from the WorldClim database (Hijmans, Cameron, Parra, Jones, & Jarvis, 2005) at a resolution of 2.5′ through DivaGIS 7.5 software. These variables were chosen because they describe the central tendency as well as the variation in the temperature and precipitation and therefore represent the physiological limits of amphibians (Buckley & Jetz, 2008; da Silva, Almeida‐Neto, et al., 2012): (1) the AMT; (2) the maximum temperature of the warmest month (MTWM); (3) the minimum temperature of the coldest month (MTCM); (4) the difference between the MTWM and MTCM; (5) the annual precipitation; (6) the precipitation seasonality; (7) the precipitation of the wettest quarter (PRWQ); (8) the precipitation of the driest quarter (PRDQ); and (9) the difference between the PRWQ and PRDQ.

### 2.4 Data analysis

#### 2.4.1 Beta diversity components

We calculated the dissimilarity in species composition between the different grains, using the additive partitioning approach proposed by Baselga (2010, 2012), in which the Jaccard dissimilarity index is decomposed into two additive components: (1) the species replacement component (βjtu), which measures the proportion of unique species in two sites pooled together if both sites are equally rich; and (2) the nestedness‐resultant component (βjne), which measures how dissimilar the sites are due to a nested pattern. It should be noted that nestedness‐resultant component is not a measure of nestedness itself, but a measure of the fraction of total dissimilarity that it is not caused by species replacement but instead by nestedness (Baselga, 2012).

#### 2.4.2 Congruence in the distribution of species replacement and nestedness values across different spatial scales

To determine if species replacement and nestedness values are similar across different SSs in Brazilian Atlantic Forest, we used generalized linear models, with a Gaussian distribution and the log link function (Figure 2a). For SS1, we compared if dissimilarity values between ponds are similar within each region. For SS2 and SS3, we compared if dissimilarity values of ponds (SS2) or sites (SS3) are similar among regions. When the dissimilarity values were different within or among regions, we compared the treatments using a post hoc Tukey test. We inspected the data graphically (e.g., q–q plots), and when necessary, prior to the analyses the data were log‐transformed to achieve normality and homoscedasticity.

#### 2.4.3 Relative importance of geographical distance and environmental descriptors in explaining the variation in beta diversity components

We reduced the multicollinearity among the environmental descriptors of the sites using principal component analysis (PCA). We then used the first two axes of the PCA (corresponding to 89% of the total variance) as the environmental descriptors in the analysis. The relative importance of geographical distance (Euclidean distance, representing the decay in similarity among the sampling units with distance; Nekola & White, 1999) and the environmental descriptors was calculated using variation partitioning analysis (Borcard, Legendre, & Drapeau, 1992). This approach partitions the total percentage of variation into unique and shared contributions of the sets of predictors. The total variation in the pairwise beta diversity components from hypotheses SS1, SS2, and SS3 was divided into four fractions: (1) the variation explained purely by geographical distance; (2) the variation explained purely by environmental descriptors; (3) the shared variation explained by environmental descriptors and geographical distance; and (4) unexplained variation (residual). The total variation in the pairwise beta diversity components from SS4 was divided into eight fractions. The first four are identical to the previous fractions, and the other four include (5) the variation explained purely by regions; (6) the shared variation explained by environmental descriptors and regions; (7) the shared variation explained by geographic distance and regions; and (8) the shared variation explained by environmental descriptors, geographical distance, and regions. We performed partial redundancy analysis with 999 Monte Carlo permutations to test significance of variation explained purely by environmental descriptors, geographical distance, and regions (Legendre & Legendre, 2012).

All analyses were performed with R 3.1.2 software (R Development Core Team, 2014) using the “betapart” (Baselga, Orme, Villeger, De Bortoli, & Leprieur, 2013) and “vegan” (Oksanen, Kindt, Legendre, & O'Hara, 2013) packages.

## 3 Results

### 3.1 Congruence in the distribution of species replacement and nestedness values across different spatial scales

**Figure 3:**
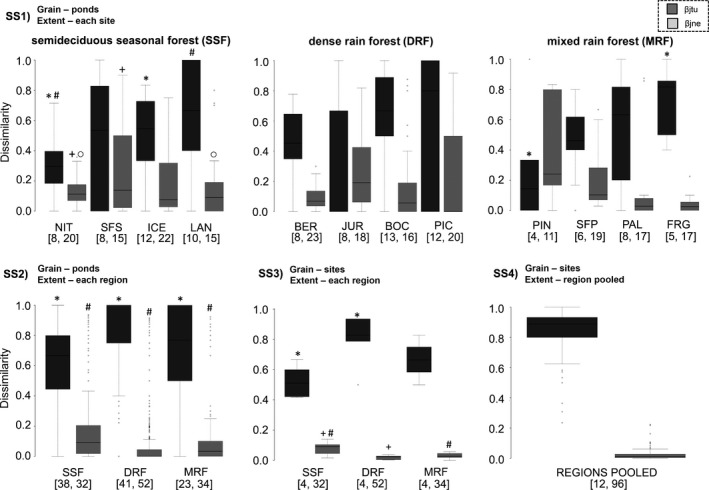
Boxplot showing the decomposition of pairwise Jaccard dissimilarity into species replacement (βjtu) and nestedness (βjne) components considering (SS1) ponds as the sampling units and each site as the extent; (SS2) ponds as the sampling units and each forest type as the extent; (SS3) sites as the sampling units and each forest type as the extent; and (SS4) sites as the sampling units and the three forest types pooled together as the extent. The horizontal line and box show the median and 50% quartiles, respectively, and the error bars display the range of the data. The numbers in brackets correspond to the quantity of the sampling units and the species richness, respectively. Similar symbols indicate significant difference (*p* < .05) among sites (SS1) or forest types (SS2 and SS3). SS, spatial scales. Legends represent the sites and forest types (semideciduous seasonal forest—SSF, dense rain forest—DRF, and mixed rain forest—MRF) described in Figure 1 and Table S1

We found that independently of SS, species replacement was the main component of the beta diversity (Figures 2b and 3). Furthermore, values of dissimilarity in species composition were different within and among regions (Figure 3): (1) For SS1, values of species replacement among ponds in NIT site were on average 1.6 times lower than other sites in SSF (*F*_3,163_ = 5.93, *p* < .001; Figure 3SS1). In MRF, values of species replacement among ponds in PIN site were on average 2.8 times lower than in FRG (*F*_3,55_ = 3.05, *p* < .03; Figure 3SS1). We did not observe difference among dissimilarity values of ponds for sites in DRF (*F*_3,208_ = 2.6, *p* > .05); (2) For SS2, we observed that increasing the extent from sites to regions, the values of species replacement among ponds increased (*p* < .001 for the three regions; Figure 3SS1,SS2). Values of species replacement (*F*_2,1814_ = 130.9, *p* < .001) and nestedness (*F*_2,1814_ = 33.5, *p* < .001) were different among regions. Ponds in DRF showed higher values of species replacement and lower values of nestedness than ponds in SSF and MRF (Figure 3SS2); (3) For SS3, we observed that increasing the grain from ponds to sites, the values of species replacement among the sampling units decreased (*p* < .001 for the regions; Figure 3SS2,SS3). Values of species replacement (*F*_2,15_ = 6.8, *p* < .01) and nestedness (*F*_2,12_ = 7.4, *p* < .01) were different among regions. Sites in SSF showed lower values of species replacement and higher values of nestedness than sites in DRF and MRF (Figure 3SS3); (4) For SS4, we observed that increasing the extent from each region to the three regions pooled together, the values of species replacement among the sites increased (*p* < .001; Figure 3SS3,SS4).

### 3.2 Relative importance of geographical distance and environmental descriptors in explaining the variation in beta diversity components

**Figure 4:**
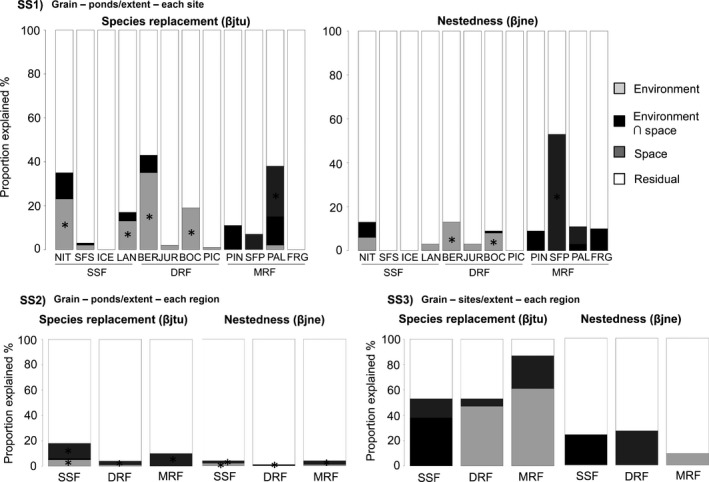
Proportion of the variation in the pairwise Jaccard dissimilarity components, species replacement (βjtu) and nestedness (βjne), explained by the correlations with environmental descriptors and geographic distance (i.e., space) considering (SS1) ponds as the sampling units and each site as the extent; (SS2) ponds as the sampling units and each forest type as the extent; and (SS3) sites as the sampling units and each forest type as the extent. Environment = variation explained purely by environment; environment ∩ space = spatially structured environment; space = variation explained purely by space. “*” indicates significant at level of .05. Legends represent the sites and forest types (semideciduous seasonal forest—SSF, dense rain forest—DRF, and mixed rain forest—MRF) described in Figure 1 and Table S1

**Figure 5:**
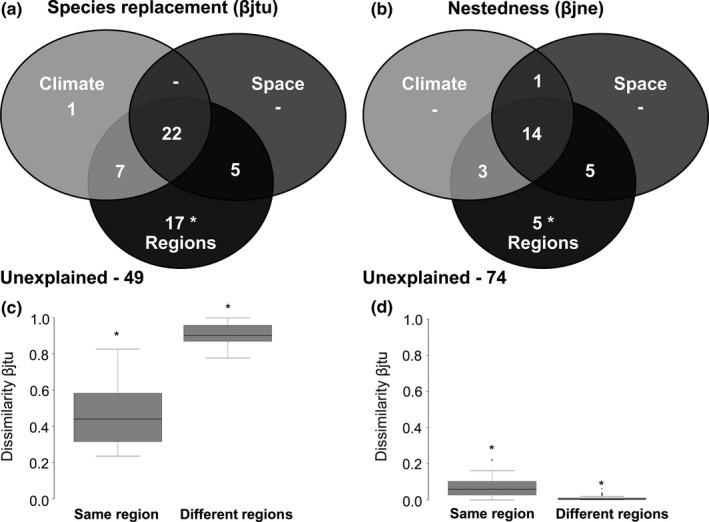
Proportion of the variation in the pairwise Jaccard dissimilarity components, species replacement (βjtu—a) and nestedness (βjne—b), explained by the correlations with climatic descriptors, geographic distance (i.e., space), and forest types considering sites as the sampling units and the three forest types (semideciduous seasonal forest, dense rain forest, and mixed rain forest) pooled together as the extent. Climate = variation explained purely by climatic descriptors; space = variation explained purely by space; regions = variation explained purely by forest types; environment ∩ space = spatially structured environment; forest types ∩ space = spatially structured forest types; climate ∩ forest types = climate together with forest types; climate ∩ forest types ∩ space = variation shared among the three descriptors, unexplained = residual. “‐” = not associated with variation in beta diversity components. Boxplot showing values of species replacement (c) and nestedness (d) components between sites in the same region and in different regions. “*” indicates significance at level of .05

The relative importance of the environmental descriptors and geographical distance within and among regions, with exception of SS1 that showed different results, was congruent (Figures 2b and 4). From the 12 sites analyzed in SS1, variation in values of species replacement for four sites was explained by environmental descriptors; for one site by geographical distance, while for seven sites was not associated with environmental descriptors or geographical distance (Figure 4SS1). Variation in values of nestedness for two sites was explained by environmental descriptors, while for ten sites was not associated with environmental descriptors or geographical distance (Figure 4SS1). For SS2, we found that variation in beta diversity components was explained by geographical distance in the three regions (Figure 4SS2). For SS3, we found that variation in beta diversity components was not explained either by climatic variables or by geographical distance (Figure 4SS3). For SS4, the regions in which the sites were located explained 17% of the variation in species replacement and 5% of the variation in nestedness (Figure 5). We observed that values of species replacement between sites in the same region were on average 0.51 lower than between sites in different regions (*F*_1,64_ = 252.5, *p* < .001; Figure 5).

## 4 Discussion

We found that independently of SS beta diversity was mainly caused by species turnover rather than the gain or loss of species. This result indicates that pattern of beta diversity is congruent within and among regions in the Brazilian Atlantic Forest. We also observed that values of species replacement and nestedness were different within and among regions. Kraft et al. (2011) showed that variation in beta diversity across broad biogeographical gradients is likely driven by difference between gamma diversity. At SS2 and SS3, values of species replacement are positively correlated with total species richness of the regions (Figure 3). On the other hand, at smallest scale (SS1) values of species replacement are not correlated with total species richness of the sites (Fig. S1). It is recognized that anuran species in SSF are less diverse, widely distributed, and have generalist reproductive modes, while anuran species in DRF are highly diverse, present small range distributions, and have specialized reproductive modes (da Silva, Almeida‐Neto, et al., 2012; Loyola, Lemes, Brum, Provete, & Duarte, 2014; Vasconcelos et al., 2014). Taken together, these results indicate that gamma diversity might influence values of beta diversity only at broad SSs (Kraft et al., 2011) and difference between beta diversity values at small scales might be associated with different processes.

The relative importance of environmental descriptors and geographical distance in explaining the variation in species replacement and nestedness, with exception of the smallest SS (SS1), was congruent among regions. It has been debated whether the explanations for community assembly and metacommunity dynamics depend on niche‐based processes (i.e., the presence and abundance of species are determined by their deterministic interactions with the abiotic and biotic environment) and/or neutral processes (presence and abundance are a result of dispersal limitation, demographic stochasticity, and random speciation). We found that at small SSs (SS1) both stochastic factors such as recruitment or random colonization (sensu Chase, 2007; Hubbell, 2001) and deterministic factors such species sorting (sensu Leibold et al., 2004) might be important mechanisms structuring anuran assemblages in ponds. These results indicate that the relative importance of each process in small SS is dependent on the studied area. Studies using ponds as sampling units have found that spatial variables explaining distribution of species composition varied from 19.8% in Dense Atlantic Forest (Provete, Gonçalves‐Souza, Garey, Martins, & Rossa‐Feres, 2014) to 10.2% in SSF (Prado & Rossa‐Feres, 2014), while environmental descriptors varied from 16.7% (Provete et al., 2014) to 21.5% (Prado & Rossa‐Feres, 2014). Therefore, we cannot generalize the associations between environmental descriptors and geographical distance obtained in one study to another when a small spatial grain is considered (Gaston et al., 2007; Lawton, 1999; Mac Nally, Fleishman, Bulluck, & Betrus, 2004; Tuomisto et al., 2016).

We observed that at the largest spatial extent (SS4), species replacement was lower among the sites within the same region than among sites among the regions. Increasing the spatial extent usually includes biogeographical regions that have undergone different processes of speciation, extinction, and colonization, resulting in different regional species pools among the regions (Barton et al., 2013; da Silva et al., 2014; Comte et al., 2016; Qian & Ricklefs, 2011). Recently, da Silva et al. (2014) showed that the distribution of taxonomic and phylogenetic anuran beta diversity at different sites in the Atlantic Forest was influenced by different biogeographical regions that experienced instable or stabile climates since the Pleistocene. Furthermore, as the SS increases, the strength of the correlation between plant communities and physiognomy may also increase (Kristiansen et al., 2012; Mac Nally et al., 2002). For example, Rueda, Rodríguez, and Hawkins (2010) and Vasconcelos et al. (2014) found that amphibian distribution patterns are not randomly distributed across space and that their distributions are broadly congruent with floristic ecoregions identified in the Atlantic Forest and Europe. Viana et al. (2016) found that biogeographical processes, acting through large‐scale environmental variation and dispersal limitation, determine the composition of aquatic plant and cladoceran communities in Europe. Thus, higher values of species replacement among sites located in different regions than among sites within the same region in the Atlantic Forest seem to have arisen from historical factors (da Silva et al., 2014) and contemporary climatic factors (da Silva, Almeida‐Neto, et al., 2012; Vasconcelos et al., 2014), restricting species distributions by means of environmental filters and/or dispersal limitations (e.g., Qian & Ricklefs, 2011; Viana et al., 2016).

Although we observed low values of environmental variables and geographical distance explaining the variation of anuran beta diversity components, they are similar to those found in other regions. Soininen (2014, 2016) performed two reviews and found that an overall mean of 26.1% (95% CI: 24.3–27.9) of the community variation was explained by environmental variables and 11% (95% CI: 10.1–11.9) was explained purely by spatial variables, respectively. We cannot ignore that there is always the possibility that important variables were not included in the analysis (Jacobson & Peres‐Neto, 2010; Soininen, 2014). For example, considering sites as sampling units and regions as extent (SS3) other variables such as percentage of native vegetation, land use and urbanization could be potential variables influencing the distribution of beta diversity at this scale. However, we highlight that our goal was not to evaluate which environmental descriptors are important to explain distribution of beta diversity, but to evaluate the congruence of the results considering the same environmental variables scales in different regions.

## 5 Conclusion

We found that, independent of the SS, species replacement was the main component of anuran beta diversity in the Brazilian Atlantic Forest. Several studies have highlighted that the ecological mechanisms driving variation in the similarity in species compositions are influenced by the effects of sampling at different spatial grains or study extents (Chase & Knight, 2013; Olivier & van Aarde, 2014; Steinbauer, Dolos, Reineking, & Beierkuhnlein, 2012). Here, we found that at small scales (ponds as the sample unit and sites as the extent), stochastic and deterministic factors might be important processes structuring anuran assemblages, indicating that the results from one study cannot be generalized to different regions (Lawton, 1999). On the other hand, at large SSs (sites as the grain and regions as the extent), the processes restricting species distributions (i.e., environmental filters and/or dispersal limitations) are more effective for drawing inferences regarding the variation in species replacement and nestedness of anurans in different regions of the Brazilian Atlantic Forest. Therefore, the consideration of multiple scales to understand the interdependence between the regional and local scales influencing the distribution of beta diversity seems to be one of the most productive avenues for future research.

Although this study was not designed to evaluate species conservation, some information obtained might be of great importance in delineating conservation plans. Ponds, independently of environmental structure, are harboring different anuran species and contribute to regional diversity. Thus, at small extent (SS1 and SS2), the conservation of anurans should focus on keeping different types of ponds. On the other hand, at broad scales (SS4) we observed that different regions in Atlantic Forest contain different species composition. In this case, beta diversity indexes seem to be a potential approach to guide spatial conservation planning based on regional species pool.

## Conflict of Interest

None declared.
