# Supplementary material for: Evaluating multiple spatial scales to understand the distribution of anuran beta diversity in the Brazilian Atlantic Forest
Source: Ecol Evol. 2017 Mar 12;7(7):2403–13. doi: 10.1002/ece3.2852 (PMC5383494; doi:10.1002/ece3.2852)
Supplement: Supplementary file 1 [file ECE3-7-2403-s001.doc]

**SUPPORTING INFORMATION**

**Evaluating multiple spatial scales to understand the distribution of anuran beta diversity in the Brazilian Atlantic Forest**

Lara Gomes Melchior, Denise de Cerqueira Rossa-Feres and Fernando Rodrigues da Silva

*Ecology and Evolution*

**Table S1.** Description of 12 Brazilian Atlantic Forest sites used in this study. All data were extracted from the literature and project database with permission of use provided by the collectors. DRF = dense rain forest; MRF = mixed rain forest; SSF = semideciduous seasonal forest.

| **Region based on vegetation** | **Municipalities** | **Number of ponds** | **Total Species richness (lowest and highest SR in the ponds)** | **Geographic coordinates** | **Sampling period** | **Sampling effort in each pond** | **References** |
| --- | --- | --- | --- | --- | --- | --- | --- |
| **DRF** | Bertioga (BER) | 8 | 23 (7-12) | Latitude: -23.7198 / Longitude: -45.8699 | 2006-2007 | 13 days | Dias (2008) |
| **DRF** | Bocaína (BOC) | 13 | 16 (1-8) | Latitude: -24.1986 / Longitude: -46.9394 | 2008-2009 | 12 days | Provete (2010) |
| **DRF** | Jureia (JUR) | 8 | 18 (2-11) | Latitude: -24.4444 / Longitude: -47.0903 | 2010 | 3 days | SISBIOTA |
| **DRF** | Picinguaba (PIC) | 12 | 20 (1-12) | Latitude: -23.3523 / Longitude: -44.8533 | 2011-2012 | 7 days | Jordani (2013) |
| **MRF** | São Francisco de Paula (SFP) | 6 | 19 (4-13) | Latitude: -29.4469 / Longitude: -50.5791 | 2005-2006 | 12 days | Both et al (2008) |
| **MRF** | Fazenda Rio Grande (FRG) | 5 | 17 (4-8) | Latitude: -25.65 /  Longitude: -49.3 | 2002-2003 | 15 days | Conte & Rossa-Feres (2007) |
| **MRF** | Palmas (PAL) | 8 | 17 (1-8) | Latitude: -16.4833 / Longitude: -51.9833 | 2008 | 13 days | Conte (2010) |
| **MRF** | São José dos Pinhais (PIN) | 4 | 11 (1-9) | Latitude: -25.5167 / Longitude: -49.1833 | 2007 | 13 days | Conte & Rossa-Feres (2006) |
| **SFS** | Santa Fé do Sul (SFS) | 8 | 15 (1-10) | Latitude: -20.2111 / Longitude: -50.926 | 2003-2004 | 18 days | Santos et al. (2007) |
| **SSF** | Nova Itapirema (NIT) | 8 | 20 (9-16) | Latitude: -21.0781 / Longitude: -49.5389 | 2003-2004 | 24 days | Vasconcelos & Rossa-Feres (2005) |
| **SSF** | Icém (ICE) | 12 | 21 (3-12) | Latitude: -20.3664 / Longitude: -49.2567 | 2004-2005 | 19 days | da Silva et al. (2012) |
| **SSF** | Luis Antônio (LAN) | 10 | 15 (2-10) | Latitude: -21.5783 / Longitude: -47.7864 | 2009-2010 | 12 days | Corrêa-Filho (2013) |

**Table S2. S**pecies list of 96 anurans recorded in the 12 Brazilian Atlantic Forest sites used in this study. * Anuran species that occur in all three regions.

|  | Semideciduous Seasonal Forest | | | | Dense Rain Forest | | | | Mixed Rain Forest | | | |
| --- | --- | --- | --- | --- | --- | --- | --- | --- | --- | --- | --- | --- |
|  | ICE | LAN | NIT | SFS | BER | BOC | JUR | PIC | FRG | SFP | PAL | PIN |
| *Aplastodiscus albosignatus* | 0 | 0 | 0 | 0 | 0 | 0 | 0 | 0 | 1 | 0 | 0 | 1 |
| *Aplastodiscus eugenioi* | 0 | 0 | 0 | 0 | 0 | 0 | 0 | 1 | 0 | 0 | 0 | 0 |
| *Aplastodiscus leucopygeus* | 0 | 0 | 0 | 0 | 1 | 0 | 0 | 0 | 0 | 0 | 0 | 0 |
| *Aplastodiscus perviridis* | 0 | 0 | 0 | 0 | 0 | 1 | 0 | 0 | 0 | 1 | 0 | 0 |
| *Bokermannohyla ahenea* | 0 | 0 | 0 | 0 | 0 | 1 | 0 | 0 | 0 | 0 | 0 | 0 |
| *Bokermannohyla circumdata* | 0 | 0 | 0 | 0 | 1 | 1 | 0 | 0 | 0 | 0 | 0 | 0 |
| *Bokermanohyla hylax* | 0 | 0 | 0 | 0 | 1 | 0 | 0 | 1 | 0 | 0 | 0 | 0 |
| *Chiasmocleis abei* | 0 | 0 | 0 | 0 | 0 | 0 | 0 | 0 | 1 | 0 | 0 | 0 |
| *Chiasmocleis albopunctata* | 0 | 1 | 0 | 0 | 0 | 0 | 0 | 0 | 0 | 0 | 0 | 0 |
| *Chiasmocleis carvalhoi* | 0 | 0 | 0 | 0 | 0 | 0 | 0 | 1 | 0 | 0 | 0 | 0 |
| *Chiasmocleis leucosticta* | 0 | 0 | 0 | 0 | 1 | 0 | 0 | 0 | 0 | 0 | 0 | 0 |
| *Chiasmocleis mantiqueira* | 0 | 0 | 0 | 0 | 0 | 1 | 0 | 0 | 0 | 0 | 0 | 0 |
| *Crossodactylus dispar* | 0 | 0 | 0 | 0 | 1 | 0 | 0 | 0 | 0 | 0 | 0 | 0 |
| *Dendropsophus berthalutzae* | 0 | 0 | 0 | 0 | 1 | 0 | 0 | 1 | 0 | 0 | 0 | 0 |
| *Dendropsophus elegans* | 0 | 0 | 0 | 0 | 1 | 0 | 1 | 1 | 0 | 0 | 0 | 0 |
| *Dendropsophus elianeae* | 1 | 0 | 0 | 0 | 0 | 0 | 0 | 0 | 0 | 0 | 0 | 0 |
| *Dendropsophus microps* | 0 | 0 | 0 | 0 | 1 | 1 | 1 | 0 | 1 | 1 | 0 | 1 |
| *Dendropsophus minutus** | 1 | 1 | 1 | 0 | 0 | 1 | 0 | 1 | 1 | 1 | 1 | 1 |
| *Dendropsophus nahdereri* | 0 | 0 | 0 | 0 | 0 | 0 | 0 | 0 | 0 | 0 | 1 | 0 |
| *Dendropsophus nanus* | 1 | 1 | 1 | 1 | 0 | 0 | 0 | 0 | 0 | 0 | 0 | 0 |
| *Dendropsophus werneri* | 0 | 0 | 0 | 0 | 0 | 0 | 1 | 0 | 0 | 0 | 0 | 0 |
| *Dermatonotus muelleri* | 1 | 0 | 0 | 1 | 0 | 0 | 0 | 0 | 0 | 0 | 0 | 0 |
| *Elachistocleis bicolor* | 0 | 0 | 1 | 0 | 0 | 0 | 0 | 0 | 1 | 0 | 0 | 0 |
| *Elachistocleis cesarii* | 1 | 0 | 1 | 1 | 0 | 0 | 0 | 0 | 0 | 0 | 0 | 0 |
| *Elachistocleis ovalis* | 0 | 0 | 0 | 0 | 0 | 0 | 0 | 1 | 0 | 0 | 0 | 0 |
| *Hypsiboas albomarginatus* | 0 | 0 | 0 | 0 | 1 | 0 | 1 | 1 | 0 | 0 | 0 | 0 |
| *Hypsiboas albopunctatus* | 1 | 1 | 1 | 1 | 0 | 0 | 0 | 0 | 0 | 0 | 0 | 0 |
| *Hypsiboas bandeirantes* | 0 | 0 | 0 | 0 | 0 | 1 | 0 | 0 | 0 | 0 | 0 | 0 |
| *Hypsiboas bischoffi* | 0 | 0 | 0 | 0 | 1 | 0 | 0 | 0 | 1 | 1 | 0 | 1 |
| *Hypsiboas faber** | 0 | 1 | 0 | 0 | 1 | 0 | 1 | 1 | 1 | 1 | 1 | 1 |
| *Hypsiboas leptolineatus* | 0 | 0 | 0 | 0 | 0 | 0 | 0 | 0 | 0 | 1 | 0 | 0 |
| *Hypsiboas lundii* | 0 | 1 | 0 | 0 | 0 | 0 | 0 | 0 | 0 | 0 | 0 | 0 |
| *Hypsiboas pardalis* | 0 | 0 | 0 | 0 | 1 | 0 | 0 | 0 | 0 | 0 | 0 | 0 |
| *Hypsiboas polytaeniae* | 0 | 0 | 0 | 0 | 1 | 0 | 0 | 0 | 0 | 0 | 0 | 0 |
| *Hypsiboas prasinus* | 0 | 0 | 0 | 0 | 0 | 0 | 0 | 0 | 1 | 0 | 0 | 1 |
| *Hypsiboas raniceps* | 0 | 0 | 1 | 1 | 0 | 0 | 0 | 0 | 0 | 0 | 0 | 0 |
| *Hypsiboas semilineatus* | 0 | 0 | 0 | 0 | 0 | 0 | 1 | 0 | 0 | 0 | 0 | 0 |
| *Itapotihyla langsdorf* | 0 | 0 | 0 | 0 | 0 | 0 | 1 | 0 | 0 | 0 | 0 | 0 |
| *Leptodactylus furnarius* | 1 | 0 | 0 | 0 | 0 | 1 | 0 | 0 | 0 | 0 | 0 | 0 |
| *Leptodactylus fuscus* | 1 | 1 | 1 | 1 | 0 | 0 | 0 | 0 | 0 | 0 | 0 | 0 |
| *Leptodactylus labyrinthicus* | 0 | 1 | 1 | 0 | 0 | 0 | 0 | 0 | 0 | 0 | 0 | 0 |
| *Leptodactylus mystaceus* | 1 | 1 | 1 | 0 | 0 | 0 | 0 | 0 | 0 | 0 | 0 | 0 |
| *Leptodactylus notoaktites* | 0 | 0 | 0 | 0 | 0 | 0 | 0 | 0 | 1 | 0 | 0 | 0 |
| *Leptodactylus plaumanni* | 0 | 0 | 0 | 0 | 0 | 0 | 0 | 0 | 0 | 1 | 1 | 0 |
| *Leptodactylus podicipinus* | 1 | 1 | 1 | 1 | 0 | 0 | 0 | 0 | 0 | 0 | 0 | 0 |
| *Leptodactylus latrans** | 0 | 0 | 1 | 0 | 0 | 0 | 1 | 1 | 0 | 1 | 1 | 1 |
| *Odontophrynus americanus* | 0 | 0 | 0 | 0 | 0 | 0 | 0 | 0 | 0 | 0 | 1 | 0 |
| *Odontophrynus cultripes* | 0 | 1 | 0 | 0 | 0 | 0 | 0 | 0 | 0 | 0 | 0 | 0 |
| *Phyllomedusa distincta* | 0 | 0 | 0 | 0 | 0 | 0 | 1 | 0 | 0 | 1 | 0 | 0 |
| *Phyllomedusa rohdei* | 0 | 0 | 0 | 0 | 1 | 0 | 0 | 0 | 0 | 0 | 0 | 0 |
| *Phyllomedusa tetraploidea* | 0 | 0 | 0 | 0 | 0 | 0 | 0 | 0 | 0 | 0 | 1 | 0 |
| *Physalaemus atlanticus* | 0 | 0 | 0 | 0 | 0 | 0 | 0 | 1 | 0 | 0 | 0 | 0 |
| *Physalaemus avelinoi* | 0 | 0 | 0 | 0 | 0 | 0 | 0 | 0 | 1 | 0 | 0 | 0 |
| *Physalaemus barrioi* | 0 | 0 | 0 | 0 | 0 | 1 | 0 | 0 | 0 | 0 | 0 | 0 |
| *Physalaemus centralis* | 0 | 0 | 0 | 1 | 0 | 0 | 0 | 0 | 0 | 0 | 0 | 0 |
| *Physalaemus cuvieri** | 1 | 1 | 1 | 1 | 0 | 0 | 1 | 0 | 1 | 1 | 1 | 1 |
| *Physalaemus gracilis* | 0 | 0 | 0 | 0 | 0 | 0 | 0 | 0 | 0 | 1 | 1 | 1 |
| *Physalaemus marmoratus* | 1 | 0 | 1 | 1 | 1 | 0 | 0 | 0 | 0 | 0 | 0 | 0 |
| *Physalaemus nattereri* | 1 | 0 | 0 | 1 | 0 | 0 | 0 | 0 | 0 | 0 | 0 | 0 |
| *Physalaemus olfersii* | 0 | 0 | 0 | 0 | 1 | 1 | 0 | 0 | 1 | 0 | 0 | 0 |
| *Proceratophrys boiei* | 0 | 0 | 0 | 0 | 0 | 0 | 0 | 0 | 1 | 0 | 0 | 1 |
| *Proceratophrys melanopogon* | 0 | 0 | 0 | 0 | 0 | 1 | 0 | 0 | 0 | 0 | 0 | 0 |
| *Pseudis cardosoi* | 0 | 0 | 0 | 0 | 0 | 0 | 0 | 0 | 0 | 1 | 0 | 0 |
| *Pseudis paradoxa* | 0 | 0 | 1 | 0 | 0 | 0 | 0 | 0 | 0 | 0 | 0 | 0 |
| *Pseudopaludicola falcipes* | 1 | 0 | 0 | 0 | 0 | 0 | 0 | 0 | 0 | 0 | 0 | 0 |
| *Pseudopaludicola mystacalis* | 1 | 0 | 1 | 0 | 0 | 0 | 0 | 0 | 0 | 0 | 0 | 0 |
| *Pseudopaludicola ternetzi* | 1 | 0 | 0 | 0 | 0 | 0 | 0 | 0 | 0 | 0 | 0 | 0 |
| *Rhinella icterica* | 0 | 0 | 0 | 0 | 1 | 0 | 0 | 0 | 0 | 1 | 1 | 0 |
| *Rhinella onata* | 0 | 1 | 0 | 0 | 1 | 0 | 1 | 1 | 0 | 0 | 0 | 0 |
| *Rhinella schneideri* | 1 | 0 | 1 | 1 | 0 | 1 | 0 | 0 | 0 | 0 | 0 | 0 |
| *Scinax alter* | 0 | 0 | 0 | 0 | 0 | 0 | 1 | 1 | 0 | 0 | 0 | 0 |
| *Scinax argyreonatus* | 0 | 0 | 0 | 0 | 0 | 0 | 1 | 1 | 0 | 0 | 0 | 0 |
| *Scinax aromathyella* | 0 | 0 | 0 | 0 | 0 | 0 | 0 | 0 | 0 | 1 | 1 | 0 |
| *Scinax brieni* | 0 | 0 | 0 | 0 | 1 | 0 | 0 | 0 | 0 | 0 | 0 | 0 |
| *Scinax catharinae* | 0 | 0 | 0 | 0 | 0 | 0 | 0 | 0 | 1 | 1 | 0 | 0 |
| *Scinax crospedospilus* | 0 | 0 | 0 | 0 | 1 | 1 | 0 | 0 | 0 | 0 | 0 | 0 |
| *Scinax duartei* | 0 | 0 | 0 | 0 | 0 | 1 | 0 | 0 | 0 | 0 | 0 | 0 |
| *Scinax fuscomarginatus* | 1 | 0 | 1 | 0 | 0 | 0 | 0 | 0 | 0 | 0 | 0 | 0 |
| *Scinax fuscovarius* | 1 | 1 | 1 | 1 | 0 | 0 | 0 | 0 | 1 | 1 | 1 | 0 |
| *Scinax granulatus* | 0 | 0 | 0 | 0 | 0 | 0 | 0 | 0 | 0 | 1 | 1 | 0 |
| *Scinax hayii* | 0 | 0 | 0 | 0 | 1 | 0 | 1 | 1 | 0 | 0 | 0 | 0 |
| *Scinax hiemalis* | 0 | 0 | 0 | 0 | 1 | 0 | 0 | 0 | 0 | 0 | 0 | 0 |
| *Scinax littoraris* | 0 | 0 | 0 | 0 | 0 | 0 | 1 | 1 | 0 | 0 | 0 | 0 |
| *Scinax obtriangulatus* | 0 | 0 | 0 | 0 | 0 | 1 | 0 | 0 | 0 | 0 | 0 | 0 |
| *Scinax perereca* | 0 | 0 | 0 | 0 | 0 | 0 | 1 | 1 | 1 | 1 | 1 | 1 |
| *Scinax perpusillus* | 0 | 0 | 0 | 0 | 0 | 0 | 0 | 1 | 0 | 0 | 0 | 0 |
| *Scinax rizibilis* | 0 | 0 | 0 | 0 | 0 | 0 | 0 | 0 | 1 | 0 | 0 | 0 |
| *Scinax ruber* | 0 | 0 | 0 | 0 | 1 | 0 | 0 | 0 | 0 | 0 | 0 | 0 |
| *Scinax similis* | 1 | 1 | 1 | 1 | 0 | 0 | 0 | 0 | 0 | 0 | 0 | 0 |
| *Scinax squalirostris* | 0 | 0 | 0 | 0 | 0 | 1 | 0 | 0 | 0 | 1 | 0 | 0 |
| *Scinax trapicheiroi* | 0 | 0 | 0 | 0 | 0 | 0 | 1 | 1 | 0 | 0 | 0 | 0 |
| *Scinax uruguayus* | 0 | 0 | 0 | 0 | 0 | 0 | 0 | 0 | 0 | 0 | 1 | 0 |
| *Sphaenorynchus surdus* | 0 | 0 | 0 | 0 | 0 | 0 | 0 | 0 | 0 | 0 | 1 | 0 |
| *Trachycephalus dibernardoi* | 0 | 0 | 0 | 0 | 0 | 0 | 0 | 0 | 0 | 0 | 1 | 0 |
| *Trachycephalus mesopheus* | 0 | 0 | 0 | 0 | 0 | 0 | 1 | 1 | 0 | 0 | 0 | 0 |
| *Trachycephalus typhonius* | 1 | 0 | 1 | 1 | 0 | 0 | 0 | 0 | 0 | 0 | 0 | 0 |
| **Species richness** | **21** | **15** | **20** | **15** | **23** | **16** | **18** | **20** | **17** | **19** | **17** | **11** |

**
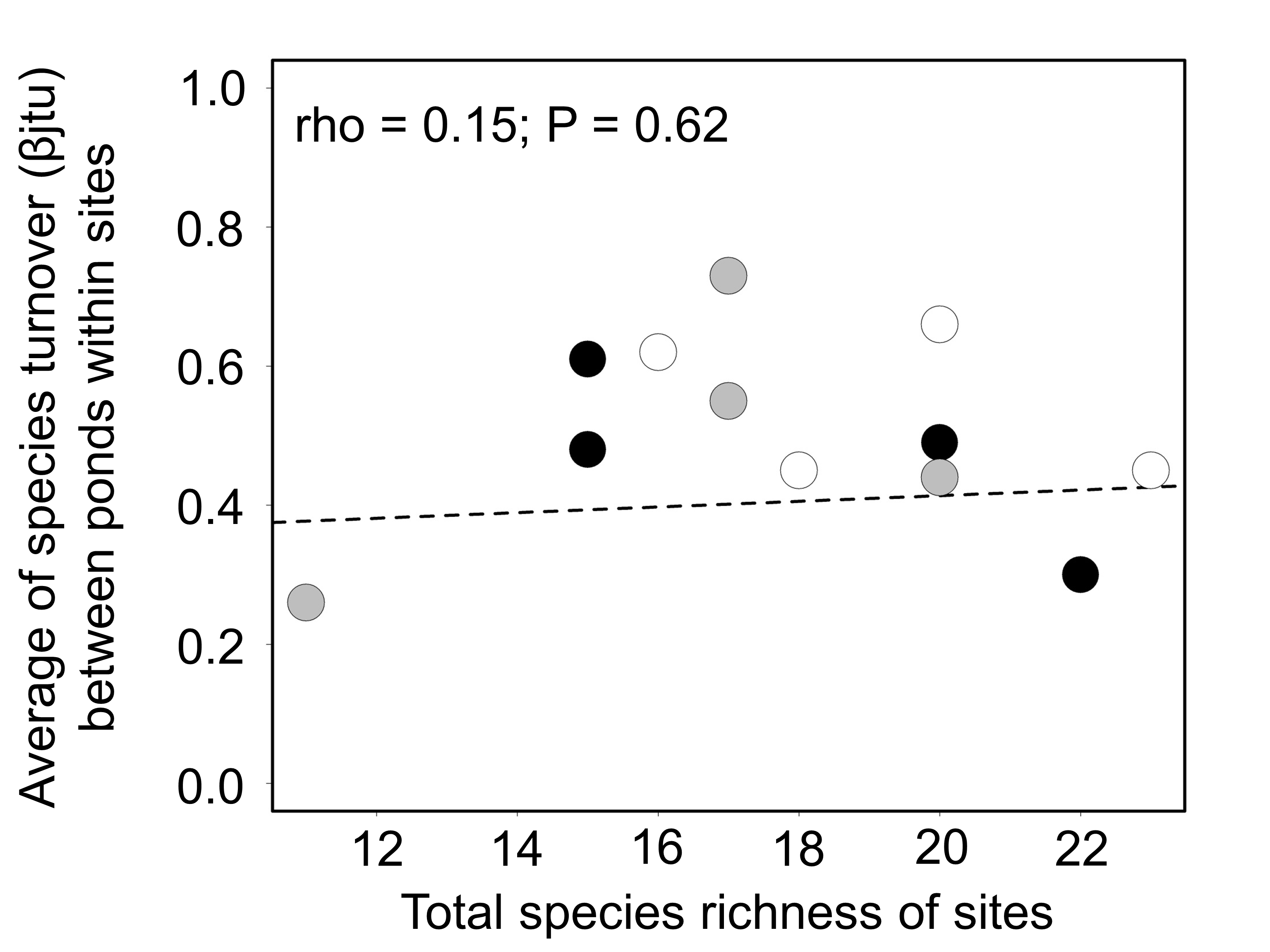
**

**
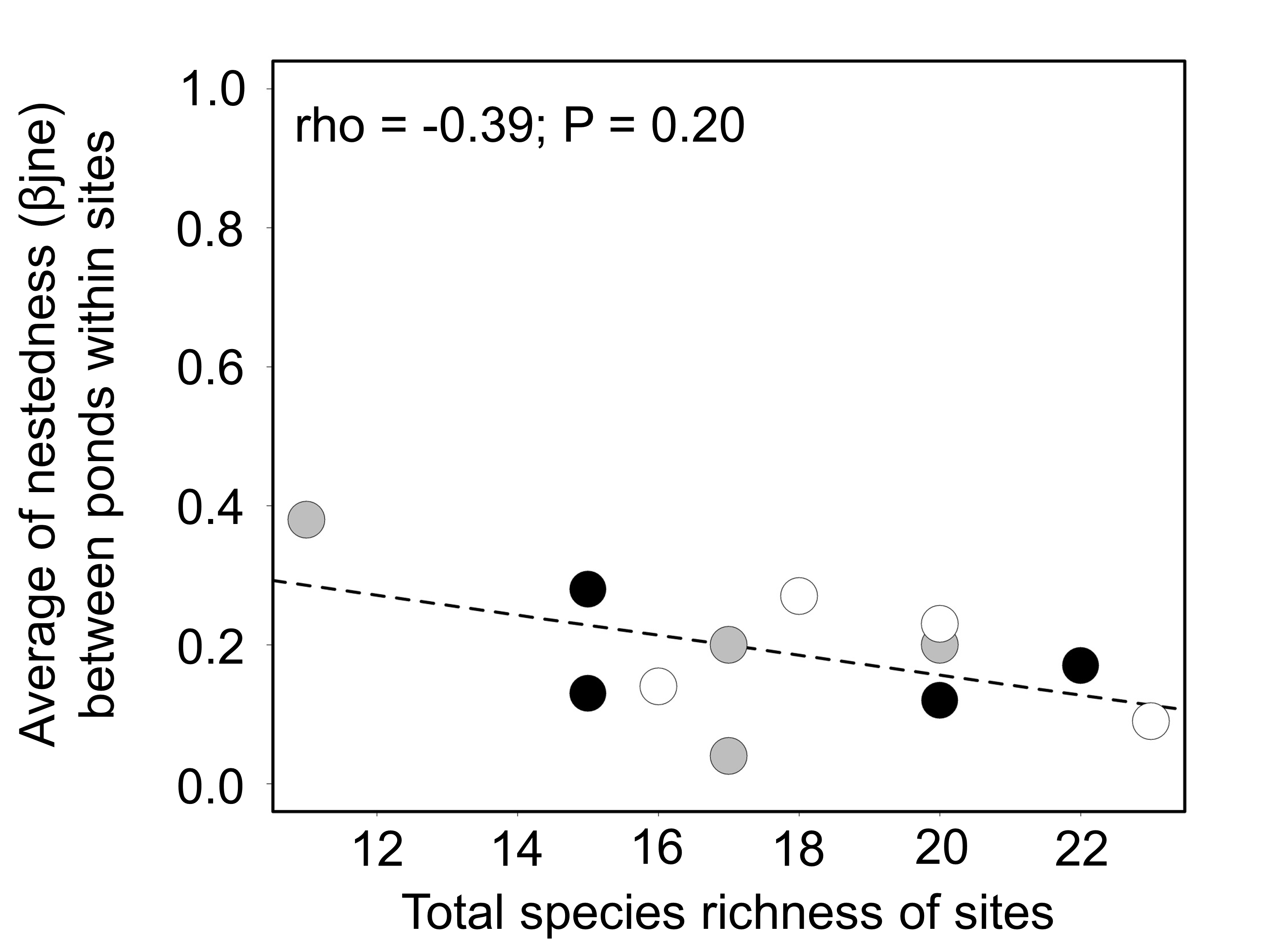
**

**Figure S1 –** Correlations between averages of species replacement (A) e nestedness (B) dissimilarities with total species richness of the sites. Different colors represent different regions. Black = Semideciduous Seasonal Forest; White = Dense Rain Forest; and Gray = Mixed Rain Forest.

**REFERENCES**

Both, C., Solé, M., Santos, T.G. & Cechin, S.Z. (2009) The role of spatial and temporal descriptors for neotropical tadpole communities in southern Brazil. [*Hydrobiologia*](http://download.springer.com/static/pdf/642/art%253A10.1007%252Fs10750-008-9685-5.pdf?auth66=1398422358_4ae0acccf4c8ffdfb9640a8378e1377e&ext=.pdf) , **624,** 125–138.

Conte, C.E. (2010) Diversidade de anfíbios da floresta com araucária. PhD thesis. Programa de Biologia Animal, Universidade Estadual Paulista, campus de São José do Rio Preto.

Conte, C.E. & Rossa-Feres, D.C. (2006) Diversidade e ocorrência temporal da anurofauna (Amphibia, Anura) em São José dos Pinhais. *Revista Brasileira de Zoologia*, **23,** 162–175.

Conte, C.E. & Rossa-Feres, D.C. (2007) Riqueza e distribuição espaço-temporal de anuros em um remanescente de Floresta de Araucária no sudeste do Paraná. *Revista Brasileira de Zoologia*, **24,** 1025–1037.

Corrêa-Filho, D.T. (2013) Estruturação de uma metacomunidade de girinos e adultos de anuros no Cerrado : influências ambientais e filogenéticas. Master thesis, Programa de Ecologia, Universidade Estadual de Campinas, Campinas.

da Silva, F.R., Candeira, C.P. & Rossa-Feres, D.C. (2012) Dependence of anuran diversity on environmental descriptors in farmland ponds. *Biodiversity and Conservation*, 21, 1411–1424.

Dias, N.Y.N. (2008) Estrutura e ecomorfologia de taxocenoses de girinos de Mata Atlântica. Master thesis, Programa de Biologia Animal, Universidade Estadual Paulista, campus de São José do Rio Preto.

Jordani, M.X. (2013) Influência de processos ecológicos e filogenéticos na estruturação de comunidades de girinos. Master thesis, Programa de Biologia Animal, Universidade Estadual Paulista, campus de São José do Rio Preto.

Provete, D.B. (2010) Uso de recursos e padrão de co-ocorrência com insetos predadores em comunidades sub-tropicais de girinos. Master thesis, Programa de Biologia Animal, Universidade Estadual Paulista, campus de São José do Rio Preto.

SISBIOTA (2010) Project of tadpole frogs from Atlantic, Amazon, Pantanal, Cerrado and transition zones: morphological, spatial distribution and diversity patterns. National System Biodiversity research (FAPESP Process 2010/52321-7 and CNPq 563075/2010-4). Responsible researcher: Denise de Cerqueira Rossa-Feres.

Santos, T.G., Rossa-Feres, D.C. & Casatti, L.(2007) Diversidade e distribuição espaço-temporal de anuros em região com pronunciada estação seca no sudeste do Brasil. *Iheringia*, **9**7, 37–49.

Vasconcelos, T.S. & Rossa-Feres, D.C. (2005) Diversidade, distribuição espacial e temporal de anfíbios anuros (Amphibia, Anura) na região noroeste do Estado de São Paulo, Brasil. *Biota Neotropica,* **5,** 137–150.
